# Immune Function of Endothelial Cells: Evolutionary Aspects, Molecular Biology and Role in Atherogenesis

**DOI:** 10.3390/ijms23179770

**Published:** 2022-08-29

**Authors:** Stanislav Kotlyarov

**Affiliations:** Department of Nursing, Ryazan State Medical University, 390026 Ryazan, Russia; skmr1@yandex.ru

**Keywords:** atherosclerosis, endothelial cells, innate immune system, hemodynamics, inflammation, lipid mediators, nitric oxide, NO, eicosanoids

## Abstract

Atherosclerosis is one of the key problems of modern medicine, which is due to the high prevalence of atherosclerotic cardiovascular diseases and their significant share in the structure of morbidity and mortality in many countries. Atherogenesis is a complex chain of events that proceeds over many years in the vascular wall with the participation of various cells. Endothelial cells are key participants in vascular function. They demonstrate involvement in the regulation of vascular hemodynamics, metabolism, and innate immunity, which act as leading links in the pathogenesis of atherosclerosis. These endothelial functions have close connections and deep evolutionary roots, a better understanding of which will improve the prospects of early diagnosis and effective treatment.

## 1. Introduction

Despite a rather long period of intensive study, atherosclerosis is still a serious clinical problem [1]. Atherosclerotic cardiovascular diseases are one of the key causes of health care seeking, hospitalizations, temporary and permanent disability, and mortality in many countries of the world [2,3,4]. The economic and social burden associated with atherosclerosis, both globally and for individual patients, is considered to be so significant that it makes atherosclerosis the leading medical problem of our time [5,6,7]. It is important to note that atherosclerotic cardiovascular diseases are quite often diagnosed at clinically advanced stages, when therapeutic options are already limited and do not allow the achievement of a cure for all patients. These and other data reinforce the understanding of the clinical relevance of exploring new details of the mechanisms of atherosclerosis development. The transition to personalized therapy based on an understanding of the individual trajectory of the disease appears to be an important direction in future cardiology, which will improve treatment efficacy in the long term.

Atherosclerosis is predominantly prevalent in older age groups, especially those with risk factors such as overweight and obesity, low physical activity, smoking, and dyslipidemia, as well as a number of comorbid conditions such as arterial hypertension, chronic obstructive pulmonary disease (COPD), and diabetes [8,9]. Correction of risk factors is considered the most important therapeutic task, both at the stage of prevention and as part of the scheme of treatment of patients [10]. It allows for the reduction of the rate of progression of atherosclerosis and its clinical manifestation.

It is important to note that despite the systemic nature of the key risk factors, the progression of atherosclerosis in the arterial bed is not diffuse, but more characteristic of certain areas of the arteries which have bends and branches. Such sites include coronary arteries, carotid artery bifurcations, and branches of lower limb arteries, in which local hemodynamic factors act [11].

A growing body of evidence confirms the involvement of the endothelium in the early stages of atherosclerotic lesions, when endothelial dysfunction promotes the adhesion of immune cells circulating in the bloodstream, which initiate further stages of atherosclerosis progression [12]. These and other data have strengthened the understanding of the role of endothelial cells in atherogenesis. The endothelial cells lining all blood vessels in a monolayer are at the blood–tissue interface, forming this interface and regulating its permeability.

**Figure 1:**
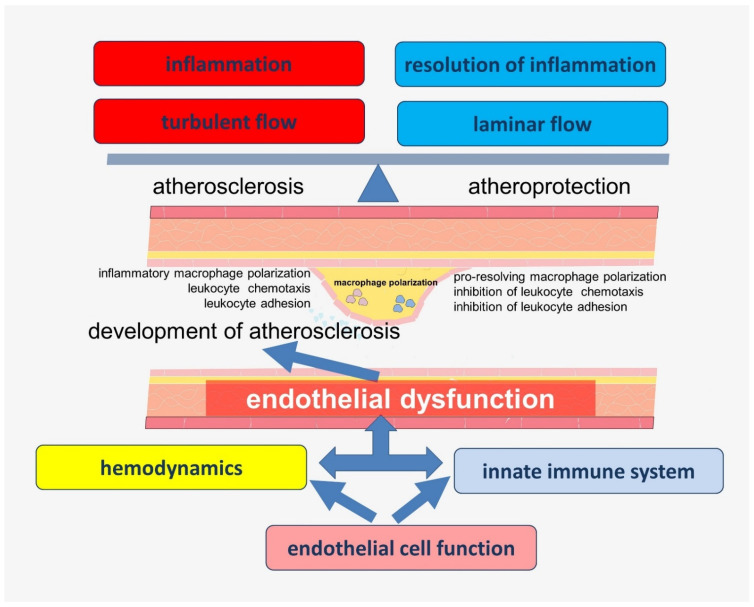
The significance of hemodynamic disturbances and inflammation in the development of atherosclerosis.

The data obtained in recent years have significantly expanded the understanding of the functions of endothelial cells, which allows them to be considered as a key participant of vascular biology. Many of these functions are closely related to each other and have complex pathways of regulation. Endothelial dysfunction associated with impaired nitric oxide (NO) bioavailability is considered to be one of the key links in the early history of atherogenesis. In addition, inflammation in the vascular wall, which is associated with an imbalance in the production of lipid mediators involved in the activation and resolution of inflammation, is another key step determining the rate of progression of atherosclerosis [13]. In addition, local changes in vascular hemodynamics, such as turbulent blood flow, may be related to the localization of atherosclerotic lesions. A growing body of evidence supports the idea that these chains of pathogenesis are closely linked (Figure 1), but the keys to understanding many of these connections are still not available to researchers and clinicians.

A better understanding of some of these functions has come from the analysis of data obtained in experimental animal models, which has also further expanded concepts about the evolutionary roots of endothelial function and added to some gaps in the interpretation of the pathophysiological mechanisms of atherogenesis. Thus, the aim of this review is to discuss the role of evolutionarily determined molecular mechanisms in atherogenesis underlying the cross-linkages of endothelial cell functions in innate immunity and participation in the regulation of vascular hemodynamics.

## 2. Origin and Function of the Endothelium

Endothelial cells form the inner lining of blood vessels and play a key role in the functioning of the barrier between tissues and blood. Moreover, the endothelium is phenotypically specialized for different tissue types. In the brain and retina, endothelial cells form specialized tight junctions that ensure the functioning of the histo-hematic barrier against the penetration of circulating molecules and cells into these tissues. On the contrary, in the liver and kidneys, i.e., the organs providing filtration functions, the endothelium may be discontinuous, which promotes infiltration and extravasation of circulating molecules and particles in the bloodstream [14].

Thus, the vascular endothelium is characterized by heterogeneity and plasticity, which are the driving forces behind the versatility of endothelial cells in matching the unique physiological function of each organ.

The origin of endothelial cells, as well as their relationship with other cells of the vascular wall and cells circulating in the bloodstream, is the subject of intense debate. During embryogenesis, the population of hemogenic endothelial cells, which is located in the aorta–gonad–mesonephros (AGM) region of the embryo, transforms into hematopoietic stem cells in a process called endothelial–hematopoietic transition (EndHT) [15,16]. The possibility of transition of endothelial cells into hematopoietic stem cells (rEC-HSCs) has also been shown in adult mice [17].

A factor of importance in atherogenesis is the endothelial–mesenchymal transition (EndMT). Apoe^−/−^ mice that were on a high-fat diet have been shown to develop atherosclerosis with intense EndMT development [18]. It is thought that EndMT may lead to the transdifferentiation of endothelial cells into mesenchymal cell types, such as smooth muscle cell-like and fibroblast-like cells [19]. Meanwhile, a growing body of evidence suggests the importance of vascular smooth muscle cells (VSMCs) in the progression of atherosclerosis. EndMT may contribute to atherogenesis by enhancing inflammation in the area of atherosclerotic lesions, leading to progressive growth of atherosclerotic plaque. In addition, EndMT contributes to cardiac fibrosis [20].

It is assumed that EndMT is a link between inflammation, impaired hemodynamics, and tissue remodeling leading to plaque formation [18]. This assumption is supported by data indicating that that inflammation and impaired shear stress downregulate endothelial FGFR1 expression, which in turn leads to activation of TGF-β signaling and induces EndMT [18]. Shear stress induces EndMT through the Snail transcription factor [21]. EndMT was shown to be inhibited by uniform laminar shear stress, whereas disturbed blood flow promoted EndMT and atherogenic endothelial cell differentiation in vitro and in vivo [22]. In addition, oxidized low-density lipoprotein (ox-LDL) induced EndMT in human aortic endothelial cells [23].

Heterogeneity associated with hemodynamic disturbances and reprogramming of the endothelium from atheroprotective to proatherogenic phenotypes, including EndMT, is of clinical interest. EndMT as well as EndHT and endothelial-to-immune-like cell transition (EndICLT) have been shown to cause blood flow disturbances [24]. The possibility of EndHT and EndICLT in disturbed flow is of great interest, but their role in atherogenesis is not yet clear.

A growing body of evidence shows that the endothelium has a wide range of means to regulate hemodynamics and inflammation, many of which have closely overlapping connections that may be disrupted in atherogenesis. Early events associated with atherosclerosis include activation of endothelial cells and their production of various agents that enhance leukocyte chemotaxis, followed by adhesion and extravasation. In addition, the endothelium is directly involved in the production of proinflammatory agents and also increases permeability, including through mechanisms of lipid transcytosis. Vascular wall cells are in close cooperation, participating in the co-production of lipid mediators associated with inflammation and inflammation resolution.

### Evolutionary Aspects of Endothelial Function

Analysis of the evolution of the endothelium has contributed to expanding the boundaries of our knowledge of its function. The emergence of the endothelium is thought to have occurred in vertebrates and coincided with the development of adaptive immunity and changes in some hemodynamic characteristics of blood flow, which allowed a decrease in circulating blood volume and an increase in blood pressure.

**Figure 2:**
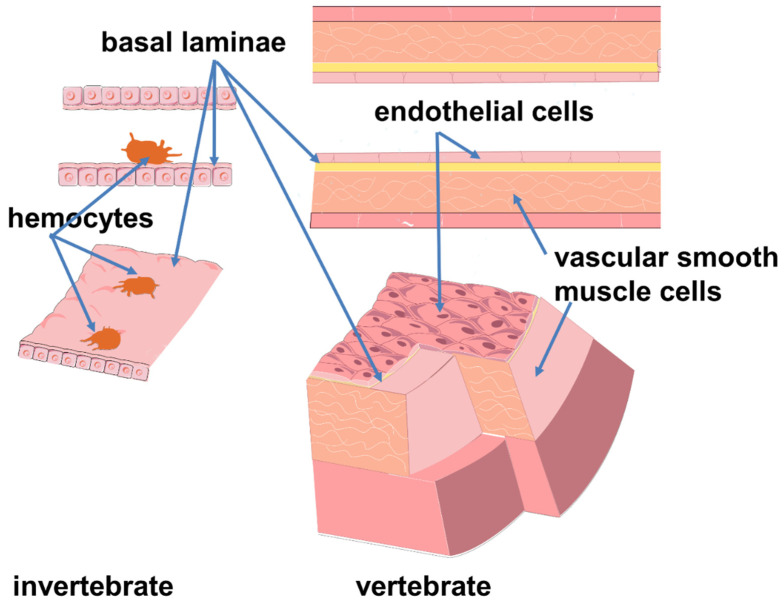
A model of invertebrate and vertebrate vessels.

It is known that most of the invertebrates, which make up the majority of biodiversity today, lack the vascular endothelial lining [25]. However, in some of them, such as cephalopods, annelids, and amphioxus, the vascular wall has cells clinging to the lumen surface [26,27,28,29,30,31]. These cells are shaped like amebocytes and do not form a continuous layer. They are not connected by the intercellular junctions typical of vertebrate endothelial cells and rarely appear attached to the basal lamina [30,32]. It is suggested that the transition between amebocytes and endothelium involved the acquisition of an epithelial phenotype, and immunological cooperation is seen as an early function of these protoendothelial cells [27]. The model of blood system transition from invertebrates to vertebrates suggests the origin of true endothelial cells from circulating (blood-like) cell progenitors (Figure 2) [33]. Thus, maintenance of blood pressure and immune function have some common connections. Although the circulatory and immune systems of invertebrates and vertebrates have profound differences, some evolutionarily conserved mechanisms underlying the cross-linkages of hemodynamic and immune regulation are of interest.

In insects, hemocytes provide innate immune protection and are similar in function to vertebrate phagocytes. However, in addition to circulating hemocytes, there are also sessile hemocytes attached to tissues in the area with the swiftest hemolymph flow [34,35]. Heart-associated hemocytes, called periostial hemocytes, have been shown to phagocytize circulating pathogens within seconds of infection. Shortly thereafter, additional hemocytes migrate to the periostial regions where hemolymph flow is maximal and enhance the phagocytosis response [34,36,37]. The periostial immune region thus contains a mixture of resident hemocytes and circulating hemocytes which settle in the periostial regions in response to infection [36]. Interestingly, periostial hemocytes produce NO, which is used both to fight bacterial infections and to regulate the heart rate [38,39,40,41]. In this case, NO mediates the integration between the immune and circulatory systems [37,42]. It has been shown that infection of the African malaria mosquito with *Escherichia coli*, *Staphylococcus aureus*, and *Staphylococcus epidermidis* increases the regulation of nitric oxide synthase in both heart and periostial hemocytes, which reduces the heart rate of mosquitoes and changes the proportional directionality of heart contractions [42]. The areas with high hemolymph flow provide ideal conditions for periostial hemocytes to destroy circulating pathogens and regulate hemodynamics [42].

Moreover, in Drosophila, NO induces an immune response through the Toll/Imd signaling pathways, including activating the production of an antimicrobial peptide [43,44,45]. When introduced into the hemocoel of *Drosophila melanogaster* larvae, NO was shown to activate the gene encoding the antimicrobial peptide diptericin [43]. Interestingly, when reinfected, hemocytes were characterized by altered phagocytic activity and increased expression of nitric oxide synthase [46]. Inhibition of nitric oxide synthase (NOS) in Drosophila increased larval sensitivity to Gram-negative bacterial infection and abolished the induction of the antimicrobial peptide diptericin [44].

In insects, the function of the uptake of soluble macromolecules and colloids is performed by pericardial cells, whereas hemocytes uptake particles [47,48,49]. The proximity of pericardial cells to periostial hemocytes is necessary for rapid uptake of pathogen breakdown products [36]. Reactive oxygen species (ROS) from pericardial cells have also been shown to act in a paracrine manner, regulating normal cardiac function in Drosophila [50]. In this case, insect pericardial cells are considered to be analogous to the reticuloendothelial system of vertebrates [51]. Amphioxus endothelial cells have been shown to possess the ability to endocytose also for the disposal of exogenous proteins [31]. It has been shown that mammalian liver sinusoidal endothelial cells can participate in the removal of various physiological and exogenous macromolecules such as polysaccharides, proteins, lipids, and nucleic acids from the blood [48]. To perform this function, liver endothelial cells express several types of specific receptors for the endocytosis of major physiological wastes, making them important nonphagocytic scavenger cells [48].

The process of migration and adhesion of circulating insect hemocytes resembles the early stages of extravasation of vertebrate leukocytes during inflammation, when activated endothelial cells produce factors that promote rolling, arresting, spreading, and diapedesis of monocytes [40,52,53,54]. It has been shown that the migration of insect hemocytes is mediated by eicosanoids [55]. At the same time, the effect of eicosanoid biosynthesis inhibitors is dose-dependent. Treatment of hemocytes with dexamethasone and indomethacin, which inhibit the cyclooxygenase (COX) and lipoxygenase (LOX) pathways, resulted in a significant decrease in their migration rate (≈42% of hemocytes) [55]. In another study, it was shown that the increase in hemocyte density in response to bacterial infection was associated with products of cyclooxygenase enzymatic activity (prostaglandin E2) [56].

It is important to note that in insects and crustaceans, circulatory currents influence immune responses, just as immune responses in vertebrates are inextricably linked to blood and lymph flow [36,37,57]. Moreover, the insect immune system can regulate the circulatory system [58].

Thus, the increase of blood pressure and improvement of the circulatory and immune systems are related to the development of endothelial function as a key participant integrating some evolutionarily conserved functions of the innate immune system and hemodynamic regulator.

## 3. Cross-Links in the Regulation of Hemodynamics and Innate Immunity Involving Endothelial Cells

The current concept of vascular hemodynamics assumes that peripheral vascular blood flow is laminar, i.e., blood flow moves in orderly parallel layers along the long axis of the vessel. These layers do not mix but move at different velocities—from minimal along the arterial wall to maximum in the center of the vessel. Several physical forces act on endothelial cells at the same time [59]. The force due to the effect of the boundary blood flow on the endothelium is known as shear stress and is considered one of the most important hemodynamic factors affecting endothelial cell structure and function [60]. Shear stress acts parallel to the endothelial cell surface and depends on physical factors such as blood flow velocity and viscosity, and viscosity is not constant and can also vary [61]. In the straight parts of the arterial tree, blood flow is thought to be laminar. High shear stress values correspond to this. However, the real anatomy of many vessels besides straight sections implies various bends and bifurcations, which affect the character of blood flow and shear stress values [62]. Such bends, stenoses, or branches of arteries are accompanied by the formation of disordered, chaotic turbulent blood flows [63]. Such blood flow patterns are characterized by uneven distribution of low shear stresses. It is generally accepted that laminar blood flow is considered to be physiological, while the appearance of turbulence is an important atherogenic factor [64]. There is convincing evidence linking peculiarities of vascular hemodynamics with the localization of atherosclerotic lesions [65,66,67].

Endothelial cells are able to detect changes in the character of blood flow [68]. Changes in shear stress correspond to the polarization of endothelial cells, which involves changes in cell orientation in the direction of blood flow [69,70,71]. Polarization involves changes in the arrangement of cell organelles, as well as changes in the cell cytoskeleton and the composition and structure of plasma membranes [69,70]. At the same time, endothelial cells, producing a number of biologically active substances, can affect arterial tone and, consequently, blood flow velocity and shear stress values [72]. It is important to note that the character of hemodynamics is a link in the development of inflammation in the vascular wall.

In addition, shear stress activates several transcription factors, such as Krüppel-like Factor 2 (KLF2) and KLF4, which, in turn, alter the expression of several genes and are essential for maintaining endothelial integrity and quiescence [73,74,75,76]. Laminar blood flow was found to induce KLF2 and KLF4 expression in the vascular endothelium, whereas KLF2 and KLF4 expression is reduced in areas of impaired blood flow as well as in areas prone to atherosclerosis [77,78,79,80]. Expression of KLF2 and KLF4 causes increased expression of endothelial nitric oxide synthase (eNOS) and promotes the differentiation of endothelial progenitor cells into endothelial cells [74,77,81,82]. In addition, KLF2 and KLF4 reduce the expression of inflammatory genes in the endothelium [83].

Interestingly, KLF2 and KLF4 show functional overlap in myeloid and endothelial cells, showing a similar evolutionary trajectory and relationship [75]. KLF4 in myeloid cells has been shown to be an important regulator of the early proinflammatory immune response [84]. Interestingly, in humans and mice, consumption of fatty foods reduces levels of myeloid KLF2, which is a nodal regulator of central and peripheral metabolic inflammation [85]. In turn, KLF4, through transcriptional interactions with STAT6, modulates stimulate alternative (M2) macrophage polarization [75,86,87]. It is also suggested that the KLF4/eNOS signaling pathway may be involved in the EndMT process [88].

Thus, hemodynamics and innate immunity are closely linked in the vascular wall, and the endothelium is at the crossroads of these connections and can participate in their regulation.

### 3.1. Participation of Endothelium in the Regulation of Vascular Hemodynamics

According to modern concepts, endothelial cells use several mechanisms to control vascular lumen. A decrease in arterial diameter is accompanied by an increase in shear stress. The endothelium is able to detect and respond to these changes by the production of vasodilatory agents, including NO and endothelial hyperpolarizing factor, resulting in an increase in the vascular lumen and a decrease in shear stress. Endothelium-dependent vasoconstriction is associated with angiotensin II, endothelin, and superoxide anion

#### 3.1.1. Nitric Oxide (NO)

NO is the best-known vasodilatory endothelial molecule. NO is considered a key player in the regulation of vascular hemodynamics. Nanomolar concentrations of NO promote the relaxation of VSMCs, which ensures vasodilatation. NO bioavailability disorders are the subject of numerous studies that link these disorders to endothelial dysfunction. However, the regulation of vascular tone is not the only function of NO. It is also involved in the regulation of platelet and leukocyte adhesion, thrombosis, and fibrinolysis [89,90,91].

NO synthesis in the endothelium is carried out by a specific isoform of eNOS, also known as NOS3 [92]. In addition to eNOS, neuronal nitric oxide synthase (abbreviated: nNOS, NOS1, NOSI) and inducible NOS (abbreviated: iNOS, NOS2, NOSII) are also present in other cells [93,94]. In contrast to constitutive eNOS and nNOS, inducible iNOS is mainly regulated by microbial agents and cytokines, generating high levels of NO [95].

Mechanical stimulation of endothelial cells by blood flow triggers a complex cascade of biochemical reactions involving numerous cellular mechanosensors and enzymes, leading to the activation of eNOS [72]. These mechanisms include mechanosensory ion channels, G-protein-coupled receptors (GPCRs)—which are activated due to plasma membrane deformation—and stimulation of integrins by shear stress. The chain of events associated with these mechanisms is thought to include rapid calcium activation, which leads to the formation of calcium–calmodulin complexes and the subsequent recruitment of eNOS from caveolae. Subsequent phosphorylation of eNOS by PKC and AKT protein kinases leads to NO production [72].

In invertebrates, NOS activity has been linked to a variety of functions, including neurotransmission, immune responses, response to environmental stress, defense, nutrition, etc. [96]. Although the endothelial lining of the blood vessels of cephalopods does not have the intercellular junctions characteristic of vertebrates, it allows the maintenance of high blood pressure levels [97,98]. Meanwhile, NO can regulate blood pressure in cephalopods through nitric oxide synthase in peripheral nerves [99]. The regulation of cardiac activity in crustaceans is carried out by NO produced by nitric oxide synthase by the heart musculature and cardiac ganglion [100]. In addition, NO produced by the crustacean heart acts on the nerve ganglion, acting as a retrograde transsynaptic signaling molecule, which has a negative chronotropic and negative inotropic effect on heartbeat [101,102]. NO has also been shown to be a local vasodilator under conditions of hypoxia in the endothelial cells of the gills of the blue mussel, *Mytilus edulis*, which emphasizes its function in improving tissue perfusion [103].

In vertebrates, eNOS is thought to occur only in reptiles, birds, and mammals [104]. In earlier vertebrates, such as fish and amphibians, NOS3 is absent in the vascular system, but prostaglandins as well as NO derived from nitrergic vasomotor nerves are involved in maintaining vascular tone [104,105]. Improvement of the system of vascular tone regulation corresponds to an increase in the levels of systemic blood pressure, which improves tissue perfusion, including in muscle tissue, which is essential for maintaining physical activity, as well as the separation of systemic and pulmonary circulation [106].

Endothelial NO is a key agent involved in the maintenance of normal blood pressure [107]. Overexpression of eNOS in mice led to a decrease in blood pressure of about 20 mmHg and plasma cholesterol levels of about 17%. This resulted in a 40% reduction in atherosclerotic lesions [108].

High blood pressure levels are of great clinical interest because of their role in atherogenesis. Blood pressure correction is an important clinical tool to reduce the risks of adverse cardiovascular events. A study conducted at the London Zoological Garden that included analysis of the heart, aorta, and central arteries of 366 birds, 185 mammals, and 69 reptiles that died of natural causes showed that atherosclerosis was seen in 1% of reptiles, 3% of mammals, and 19% of birds analyzed [109]. These results correlate well with data on mean values of systolic blood pressure, the levels of which are highest in birds (about 140 mm Hg) and mammals (about 120 mm Hg), but significantly lower in reptiles (about 40–60 mm Hg). These blood pressure values correspond to the animals’ levels of physical activity and metabolic requirements of tissues [106,110,111].

It is important to note that high arterial pressure affects the biophysical properties of endothelial cell plasma membranes, leading to a decrease in the number of caveolae-specialized lipid microdomains, which on the one hand act as a cell surface reserve of cells and, on the other hand, are an important integrating platform on which the assembly and functioning of signaling and regulatory pathways occur [112,113]. eNOS is located in the caveolae, and caveolae regulate the function of the enzyme [114,115]. Disruption of caveolae structure can affect eNOS activation and vascular reactivity. Caveolin-1, a structural component of caveolae, is also involved in the regulation of NO production through direct interaction with eNOS [116,117].

Under normal conditions, NO is produced in small physiological amounts, providing vasorelaxation and regulation of platelet and neutrophil adhesion and aggregation; it is also involved in neurotransmission [118,119]. The progression of atherosclerosis is characterized by a decrease in eNOS expression and a significant increase in total NO synthesis by other cells, mainly due to the isoform of iNOS, which is activated by proinflammatory stimuli such as cytokines and lipopolysaccharide (LPS). iNOS produces significantly more NO compared with eNOS [95,120]. However, increased NO production in the arterial intima can have negative effects [121,122,123,124]. This is due to the cytotoxicity of NO in high concentrations. This mechanism in macrophages is an important component of immune defense against numerous pathogens. The immune mechanism involving the production of NO through iNOS is well known in fish [125,126,127]. Insects make extensive use of nitric oxide for protection against pathogens, with production by both circulating and sitting hemocytes [41]. Addition of L-arginine to the food medium causes an increase in the immunity of *D. melanogaster* larvae. At the same time, the number of hemocytes increases, and lamellocytes, which play a key role in encapsulation, are characterized by increased NO production [128]. Interestingly, however, bovine pulmonary endothelial cells infected with *Cowdria ruminantium* increase NO production, reducing the viability and infectivity of *C. ruminantium* [129]. This demonstrates the possible involvement of endothelial cells in NO production for the purpose of antibacterial protection. It has been shown that in some chronic proinflammatory conditions, such as atherosclerosis, local expression of iNOS is increased in endothelium and other cell types [121,130,131,132]. Thus, it is suggested that cytokine-induced endogenous expression and activity of iNOS may play an important role in enhancing survival and maintaining endothelial function [130].

Thus, the functional activity of eNOS ensures the production of optimal levels of NO, which has an antiatherogenic effect [133]. Disruption of this balance, as well as increased activity of iNOS, leads to the production of more than the necessary levels of NO, which has a negative effect, for example, through participation in lipid peroxidation [122,134,135,136,137]. Peroxynitrite, which is formed by the interaction of NO and superoxide anions, is an oxidizing agent that impairs endothelial function [138,139]. Peroxynitrite can also block prostacyclin synthase activity and thereby impair prostacyclin production [140].

It is important to note that NO production increases in proportion to an increase in blood flow [141]. NO production and vasodilation significantly decreased in porcine carotid and femoral arteries during reversed blood flow, which is associated with an increase in superoxide production during reversed blood flow [141]. At the same time, the main source of superoxide formation during full flow reversal is NADPH oxidase [142].

It is thought that NO released under basal conditions keeps iNOS in an inactivated state through the inhibition of NF-B signaling, which regulates iNOS transcriptional mechanisms [143]. When more NO is released, NF-κB is not inhibited by NO and may promote iNOS activation, presumably through combination with superoxide anions and peroxynitrite formation [143].

**Figure 3:**
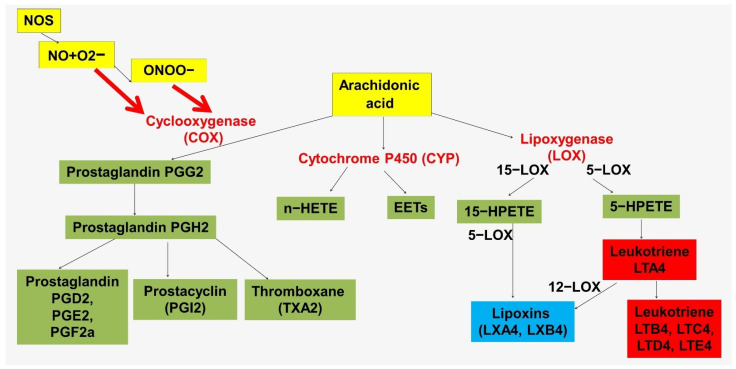
Arachidonic acid metabolic pathways and NO crosslinks. Abbreviations: EETs—epoxyeicosatrienoic acids; HETE—hydroxyicosatetraenoic acid; NOS—nitric oxide synthase; 5−HPETE—5−hydroperoxyeicosatetraenoic acid; 15−HPETE—15−hydroperoxyeicosatetraenoic acid; 5−LOX—5−lipoxygenase; 12−LOX—12−lipoxygenase; 15−LOX—15−lipoxygenase.

**Figure 4:**
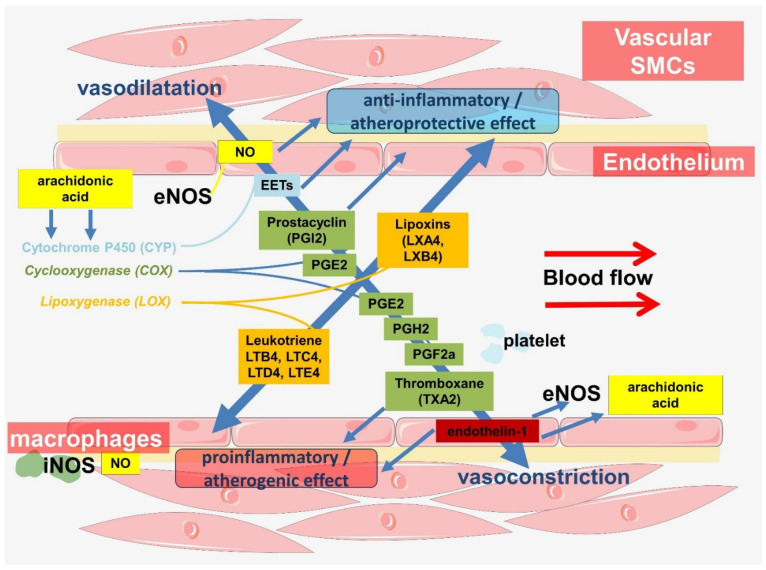
Cross-links in the regulation of hemodynamics and innate immunity involving endothelial cells.

Another mechanism linking NO and inflammation in the vascular wall is mediated by lipid mediators (Figure 3 and Figure 4). These mechanisms are highly conserved. The immune functions of NO in insects are mediated by the activation of phospholipase A2 (PLA2), which is associated with the production of proinflammatory lipid mediators [144,145]. PLA2 promotes the release of arachidonic acid from plasma membrane phospholipids.

It has been suggested that NO in insects may be an upstream link in the eicosanoid signaling pathway in response to immune challenge [146]. By increasing the activity of phospholipase A2 and eicosanoid biosynthesis, NO mediates important hemocytic immune responses [144,147]. At the same time, phospholipase A2 in insects acts as an immune regulator. It is important to note the fundamental differences in the structure of arachidonic acid in insect and mammalian membranes. In insects, arachidonic acid, which is hydrolyzed from membrane phospholipids for eicosanoid biosynthesis, is continuously replaced through the conversion of linoleic acid to arachidonic acid [148]. At the same time, in humans, arachidonic acid is a substrate for the synthesis of both proinflammatory mediators, such as leukotrienes, and specialized pro-resolving mediators, such as lipoxins (Figure 3).

Cross-signaling between NO and eicosanoids has also been shown in experiments on the mouse macrophage cell line RAW264.7 [149]. The mechanisms of this relationship may be related to the enhancement of COX activity by NO, which leads to increased production of proinflammatory prostaglandins [150]. It has been suggested that NO may interact directly with COX, for example, through S-nitrosylation, causing an increase in its enzymatic activity [149,150]. In addition to possible direct action, NO may act through the formation of peroxynitrite, reducing the effect of superoxide [143]. It should be noted that the mechanisms of these interactions are still subject to discussion [144,150,151,152]. Furthermore, the concentration of endogenous or exogenous NO plays a crucial role in the regulation of COX activity. These and other data have suggested a role for cyclooxygenase enzymes as important endogenous receptor targets for NO functions [143].

When analyzing endothelial function, it is necessary to note the importance of L-arginine, which is an important substrate for NO synthesis [153,154,155,156]. L-arginine is a conditionally essential amino acid because it can be synthesized by healthy individuals, but some conditions require additional intake of dietary arginine. Given the importance of endothelial NO, many studies have evaluated the effects of L-arginine supplementation on endothelial function [153,157]. However, the results of using such a supplement to correct endothelial dysfunction and atherosclerosis are contradictory, indicating an ambiguous role of the amino acid in cell function and a relation to the multifaceted processes occurring in the vascular wall. It was found that L-arginine can have both beneficial and adverse effects on endothelial cells, depending on the duration of its intake. On the one hand, short-term supplementation with L-arginine enhanced endothelial NO formation, and on the other hand, long-term supplementation with L-arginine induced endothelial aging associated with stimulation of mTORC1-S6K1 signaling and enhancement of arginase-II regulation [158].

L-arginine is converted to L-citrulline by eNOS to form NO. Although physiological concentrations of arginine are sufficient for eNOS, exogenous arginine affects the rate of NO synthesis. However, neither the extracellular nor intracellular concentrations determine NOS activity. This phenomenon is known as the “arginine paradox” [159,160]. VEGF can enhance eNOS activity in endothelial cells by modulating arginine transport with CAT-1 [161]. In experiments with transgenic rat blood–brain barrier (TR-BBB) endothelial cells, it was shown that argininosuccinate synthase, i.e., the enzyme that is involved in the regeneration of arginine from citrulline, plays an important role in the delivery of L-arginine to eNOS, but not iNOS [162]. Thus, extracellular L-arginine was the only source for iNOS, whereas intracellular L-arginine derived from citrulline by argininosuccinate synthase was the main source for eNOS [162].

A growing body of evidence suggests that L-arginine is an important node for the regulation of immune responses and that this function has deep evolutionary roots [163]. Experimental evidence suggests the presence of impaired lymphocyte activation in vitro when arginine is depleted [163,164]. At the same time, arginine improved wound healing and T-cell-mediated immune function [165]. Importantly, areas of inflammation are characterized by the depletion of arginine, which is utilized by immune cells [166]. Macrophages differentially use arginine depending on their function. While proinflammatory M1 macrophages preferentially metabolize arginine via iNOS, anti-inflammatory M2 macrophages preferentially metabolize it via arginase-1 to ornithine [163]. The arginase pathway provides cells with ornithine, which can be used in downstream pathways for polyamine and proline synthesis necessary for cell proliferation and tissue repair after inflammation [167]. On the other hand, the arginase pathway reduces the available L-arginine for nitric oxide synthase. Thus, different pathways of arginine metabolism in immune cells may correspond to the phase of inflammatory activity, which is related to the need for bactericidal NO or ornithine production for tissue repair after inflammation. These mechanisms have deep evolutionary roots and have also been shown to be present in invertebrate hemocytes [168,169]. In invertebrates, hemocyte arginase activity, based on the TGF-β signaling pathway, is an important component of wound healing through its involvement in collagen formation [170]. Moreover, the function of arginase as a key ornithine-producing enzyme essential for tissue repair may be evolutionarily older than the cytotoxic activity of NO [170]. Meanwhile, in vertebrates, TGF-β1 is a potent regulator of iNOS, promoting M2 activation of macrophages [170]. TGF-β is important for angiogenesis. Mice deficient in TGF-β signaling components exhibit embryonic lethality due to vascular defects [171].

It is important to note that the polarization of macrophages associated with the opposite involvement of these cells in inflammatory reactions has at its core a number of metabolic differences [166,167]. Glycolysis has been shown to promote inflammatory responses in M1 macrophages, whereas oxidative phosphorylation promotes anti-inflammatory processes and is characteristic of M2 macrophages. The switching of cellular metabolisms is thought to underlie their immune functions. In this case, arginine is required by macrophages with different polarization. Interestingly, endothelial cells as well as immune cells rely heavily on glycolysis and have lower levels of oxidative phosphorylation [172]. This metabolic strategy is known as the Warburg effect [173]. This seems all the more interesting given that endothelial cells have direct access to oxygen from the blood. All the more so, the high level of glycolysis is characteristic of quiescent endothelial cells. High glycolic activity may be a defense strategy against oxidative stress resulting from oxidative metabolism. In addition, this may be because NO competes with cellular oxygen for the terminal electron transport chain enzyme cytochrome C oxidase, thereby reducing mitochondrial respiration [174,175]. In the same way, NO generated by endothelial cells can affect immune cells by modulating their metabolism [93,176]. In addition, glycolysis can be repeatedly enhanced when necessary, as is well known from the example of immune cells. In this case, mitochondrial oxidative phosphorylation and the pentose–phosphate pathway are activated in endothelial cells as anti-inflammatory mechanisms, and increased glycolysis contributes to inflammatory responses [177]. The switching of metabolic phenotypes of endothelial cells is related to blood flow and is associated with KLF. Disrupted blood flow, characteristic of areas of atherosclerosis, has also been shown to activate glycolysis through the HIF1α/NF-kB axis [178,179]. In addition, VEGF also increases glycolysis, which has implications for angiogenesis [173]. Interestingly, in quiescent endothelial cells, glycolytic enzymes are mainly located in perinuclear regions, whereas in motile endothelial cells, glycolytic enzymes are localized with F-actin fibers in migratory cell structures, such as filopodia and lamellipodia [180].

As noted, endothelial cells express arginases, which can compete with eNOS for the substrate. Endothelial cells contain two arginase isoenzymes (arginase I and arginase II) that catalyze the same biochemical reaction but differ in their subcellular localization. Arginase activity in endothelial cells has been shown to limit NO production because of competition with NOS for the substrate. Increased arginase activity in atherogenic mice contributes to impaired NO signaling as well as increased ROS production as a result of eNOS uncoupling [181]. Arginase activity of endothelial cells may be associated with inflammation and endothelial aging as well as vascular remodeling [182]. L-ornithine, which is produced by arginase, is used to form polyamines and L-proline, which are essential for VSMCs proliferation and collagen synthesis [183]. In addition, increased levels of arginase-I increase the expression of inflammatory vascular cell adhesion molecule 1 (VCAM-1) and intercellular adhesion molecule 1 (ICAM-1), which promotes monocyte adhesion to endothelial cells and increases inflammation [182]. Increased total arginase activity, increased levels of arginase I and II in endothelial cells, and arginase II in the smooth muscle layer have been shown to be accompanied by intimal hyperplasia [184].

Exposure of the carotid artery to oscillatory flow caused more severe activation of arginase compared with unidirectional high shear stress [185]. In addition, oxLDL induced arginase II expression [186].

Interestingly, LPS induced both iNOS and arginase formation in endothelial cells, which was accompanied by increases in NO, citrulline, and urea production [187]. Meanwhile, Ng-hydroxy-L-arginine (NOHA) can accumulate in iNOS-expressing cells and is a natural inhibitor of arginase and arginase activity [187]. In addition, iNOS can S-nitrosate and activate arginase-1, which may contribute to the development of endothelial dysfunction in the aging cardiovascular system [188,189]. Both arginase isoforms have been shown to be induced by lipopolysaccharide together with iNOS in cultured macrophages [190]. The activity of iNOS and arginase is mutually regulated in macrophages by cytokines, which are necessary for NO production [191]. At the same time, low concentrations of NO protected cells from apoptosis, whereas overproduction of NO induced apoptosis [190].

As noted earlier, iNOS activity can play a protective role for endothelial cells, which is associated with NO-induced increase in expression of the anti-apoptotic protein Bcl-2, as well as NO-mediated inhibition of lipid peroxidation, which ensures the maintenance of plasma membrane integrity and function [130,192]. In this case, iNOS-dependent endothelial synthesis of NO does not contribute to cell damage, but instead is an arginine-dependent mechanism of cell protection [192]. The corresponding iNOS activity depends on the presence of L-arginine as well as on the expression and activity of cationic amino acid transporters (CAT), which are responsible for the influx of L-arginine into the cell [192]. At the same time, physiological serum concentrations of arginine do not always maintain optimal iNOS-protective activity under proinflammatory conditions in endothelial cells, and the function of endothelial iNOS may be impaired when arginine concentrations are slightly reduced [192]. This may be related to chronic inflammation, which is characterized by increased consumption of L-arginine.

Thus, the importance of L-arginine for atherogenesis is a promising area for future research. Meanwhile, NO is an important signaling molecule that is at the crossroads between the regulation of vascular hemodynamics and the innate immune response (Figure 4).

#### 3.1.2. The Significance of the Cyclooxygenase Pathway

The cyclooxygenase pathway is a source of lipid mediators associated with the regulation of vascular tone and inflammation [193]. Cyclooxygenase derivatives such as prostaglandin H2 (PGH2), prostaglandin I2 (PGI2, prostacyclin), prostaglandin E2 (PGE2), prostaglandin F2α (PGF2α), prostaglandin D2 (PGD2), and thromboxane A2 (TxA2) have been found in endothelium (Figure 3) [194]. Evidence for their involvement in the regulation of vascular tone varies, but PGI2 and PGD2 are thought to be vasodilators, whereas PGH2, PGF2α, and TxA2 are vasoconstrictors [194]. The production of these prostaglandins is regulated by many factors and also depends on age. Aging of isolated rat aortic endothelial cells has been shown to result in overexpression of eNOS, COX-1, COX-2, thromboxane synthase, hematopoietic-type prostaglandin D synthase, membrane prostaglandin E synthase-2, and prostaglandin F synthase [195]. The same study also showed increased expression of COX-1, prostacyclin synthase, thromboxane synthase, and hematopoietic-type prostaglandin D synthase in endothelial cells in hypertension [195]. Shear stress also affects gene expression and eicosanoid synthesis in vascular endothelium. Perfusion of human umbilical veins with high shear stress flow was shown to induce a significant monophasic upregulation of prostacyclin synthase and thromboxane synthase gene expression after 6 h [196]. Another study showed COX-2 induction in endothelial cells cultured under laminar shear stress conditions (10 dyne/cm^2^ for 6 h), resulting in the production of PGI2, PGE2, and PGD2 [197]. In addition, iNOS also promotes COX-2 production and oxidative stress [198].

PGI2 is formed in the endothelium of large vessels and VSMCs. By acting on the IP receptor, it promotes the relaxation of vascular smooth muscle, resulting in a pronounced vasodilator effect. An increase in blood pressure and shear stress enhances the formation of PGI2 [199]. In addition, NO has been shown to enhance the release of PGI2 from endothelial cells through the activation of cyclooxygenase [200].

Moreover, the role of PGI2 and NO in hemodynamics is age-dependent [201]. In animal experiments, it has been shown that the primary mediator produced in response to shear stress changes from PGI2 to NO from young to old age [202]. In humans, it has also been shown that the primary mediator of flow-mediated dilation of the arteries is PGI2 in youth and NO in adulthood. With the onset of coronary heart disease, the main mediator is switched to mitochondrially derived H_2_O_2_ [203]. The mediator switch of flow-induced vasodilation from NO to hydrogen peroxide in the human microcirculation may also be facilitated by ceramide [204]. This is of interest when analyzing the comorbid relationship of atherosclerosis and COPD [205]. It has also been shown that elderly people have decreased flow-mediated dilation compared with younger people, regardless of disease state [203]. These and other data support the concept of a decreased vasodilatory role of prostaglandins with age, whereas NO becomes a major endothelium-dependent mediator of vasorelaxation in adults [206].

In addition to its involvement in the regulation of hemodynamics, PGI2 suppresses platelet aggregation. In addition, PGI2 regulates the immune system with an immunosuppressive effect [207]. This effect is highly conserved. Through these and other functions, PGI2 is associated with atheroprotection. Decreased production of PGI2 in the endothelium may contribute to lipid deposition and the development of atherosclerosis [207,208].

PGE2 is a common prostaglandin, but its levels in the endothelium are lower than PGI2 [194]. It acts on EP1, EP2, EP3, and EP4 receptors, which are mainly located in VSMCs [209,210]. The vascular effects of PGE2 are different because of the opposite actions of the receptors. Activation of EP1 and EP3 leads to vasoconstriction, whereas activation of EP2 and EP4 receptors promotes vasodilation and decreases blood pressure [211]. EP1 receptor activation has been shown to increase arteriolar tone and blood pressure in type 2 diabetic mice [211]. In doing so, acting through EP4, prostaglandin E2 protects the heart from ischemia–reperfusion injury [212]. In addition, it reduces swine myocardial ischemia–reperfusion injury by increasing the expression levels of eNOS and VEGF [213]. EP2 is involved in the repair of postischemic cardiac damage by modulating macrophage recruitment [214]. At the same time, NO is required to maintain long-term expression of the COX-2 gene and sustained biosynthesis of PGE2 [215].

PGE2 modulates angiogenesis by promoting the proliferation and migration of endothelial progenitor cells [216]. In addition, PGE2 can exert both proinflammatory and anti-inflammatory effects depending on the conditions [214]. High concentrations of PGE2 inhibited cell migration, whereas low concentrations had the opposite effect [217]. Mosquito midgut cells produce and release PGE2, which attracts hemocytes to the midgut surface and enhances their patrolling activity [218]. In addition, PGE2 signaling in insects promotes antimicrobial activity through the synthesis of antimicrobial peptides [219].

PGF2α is formed in endothelial cells in small amounts, as a consequence of which it has minimal effect on endothelium-dependent contractions despite its powerful vasoconstrictor properties [194]. Laminar flow with a shear stress of 10 din/cm^2^ contributes to a decrease in PGF2α production by the endothelium compared with static conditions [197].

TXA2 is largely produced by platelets as well as by monocytes and in the endothelium and vascular smooth muscle [220,221,222,223]. TXA2 acts through the thromboxane A2 receptor, also known as the prostanoid TP receptor (TP), whose stimulation leads to activation of various signaling cascades that regulate cytoskeleton, cell adhesion, cell motility, proliferation, cell survival, and apoptosis [224]. One of the important biological actions of TXA2 is platelet activation [224]. In addition, TXA2 has a significant vasoconstrictor effect [225]. TXA2 may be associated with the development of atherosclerosis, which confirms its increased production in atherosclerosis [208,226,227]. In endothelial cells, TXA2 enhances surface expression of ICAM-1, VCAM-1, and endothelial leukocyte adhesion molecule-1 (ELAM-1) [228,229] and regulates endothelial cell migration [224,230]. At the same time, as a negative feedback regulator, TXA2 produces PGI2, which attenuates platelet aggregation and the contraction of vascular smooth muscle [224,231].

Thus, the products of the cyclooxygenase pathway in endothelial cells demonstrate multiple cross-linkages between involvement in inflammation and regulation of vascular hemodynamics.

#### 3.1.3. Endothelium-Dependent Hyperpolarizations

Epoxyeicosatrienoic acids (EETs) are formed from arachidonic acid by cytochrome P450. They are produced by endothelial cells in response to agonists such as acetylcholine and bradykinin or to physical factors such as shear stress or cyclic stretch [232,233]. Studies have identified EET as an endothelium-derived relaxing factor (EDRF).

The biological action of EET in the vascular network is related to the regulation of vascular tone, hemostasis, and inflammation. Moreover, the vasodilatory effect of EDHF is more characteristic of smaller-diameter arteries, whereas in larger vessels this role is assigned to NO [234,235]. EETs activate large-conductance calcium-activated potassium channels on vascular smooth muscles, causing them to hyperpolarize and leading to the relaxation of the arteries [236]. EETs have been shown to relax coronary arteries in an endothelium-independent manner [232]. It should be noted that in the pulmonary vasculature, EETs have a vasoconstrictor effect and may be important mediators of pulmonary hypertension [237,238].

In addition to their vasoactive function, EETs are highly active intracellular messengers that provide control of Ca2+ signaling, membrane potential, and tyrosine kinase activity [239,240]. EETs have been shown to stimulate endothelial cell growth and angiogenesis by mediating the MAPK and PI3-kinase/Akt signaling pathways and, to some extent, the eNOS pathway [241]. Stimulation of endothelial cells with VEGF has been shown to increase CYP2C expression as well as 11,12-EET levels, which are part of the physiological processes regulating angiogenesis [242]. In addition, by activating platelet hyperpolarization mediated by action on potassium channels, EETs inactivate platelets and prevent their adhesion to endothelial cells [243]. In addition, acting through GPR132, 11,12-EETs induce hematopoietic stem cell specification and enhance vertebrate hematopoiesis [244].

In addition, EETs have anti-inflammatory effects. EETs have been shown to prevent NF-κB activation and inhibit the expression by endothelial cells of cell adhesion molecules such as VCAM-1, ICAM-1, E-selectin, and monocyte chemoattractant protein-1 (MCP-1), thereby limiting monocyte adhesion to endothelium [245,246,247]. 11,12-epoxyeicosatrienoic acid was also shown to attenuate prostaglandin E2 synthesis in lipopolysaccharide-stimulated rat monocytes via COX-2 [248]. In turn, 14,15-epoxyeicosatrienoic acid decreases prostaglandin E2 production in vascular smooth muscle cells by inhibiting PGH synthase activity [249]. Meanwhile, the vasoactivity of 5,6-EET in rabbit kidneys was associated with the release of the prostaglandins PGE2 and PGI2 and the metabolism of 5,6-EET to a prostaglandin analog (5,6-epoxy-PGE1) [250]. Interestingly, in insects at the larval stage, 5,6-EET, 8,9-EET, 11,12-EET, and 14,15-EET were found in immune-associated tissues such as the adipose body and hemocytes [251].

Epoxyeicosatrienoic acids can be incorporated into and released from endothelial phospholipids, resulting in potentiation of endothelium-dependent relaxation [252]. In addition to incorporation into cellular lipids, EET can be converted by epoxide hydrolases into dihydroxyeicosatrienoic acid (DHET), which is incorporated into phospholipids to a lesser extent than EET [253]. Thus, endothelial epoxide hydrolases may play an important role in regulating endothelial function [253]. Given that DHETs are generally less biologically active, epoxide hydrolases reduce the beneficial effects of EETs [254].

Thus, EETs exhibit many biological functions that contribute in important ways to the maintenance of cardiovascular homeostasis. They limit leukocyte adhesion and transmigration through the endothelium, inhibit platelet aggregation, promote fibrinolysis, reduce VSMCs proliferation, act as PPAR agonists, and regulate various physiological processes [255,256].

#### 3.1.4. Endothelin-1

Endothelin-1 (ET-1) is the best-known isoform of the endothelin family. It is a powerful vasoconstrictor peptide and plays an important role in the regulation of vascular hemodynamics [257]. Endothelin-1 is produced by various cells, including the endothelium. Factors that stimulate ET-1 release include low shear stress, hypoxia, adrenaline, angiotensin-II, endotoxin, tumor necrosis factor-alpha (TNF-α), interleukin (IL)-1, and IL-6 [258]. In turn, high shear stress, NO, and prostacyclin inhibit ET-1 release [258,259].

Two types of endothelin receptors are known: endothelin A receptors (ETA) and endothelin B receptors (ETB), which belong to the G-protein coupled receptor family. ETA receptors located in VSMCs mediate increases in intracellular calcium concentrations and vasoconstriction [260]. In the heart, these receptors are responsible for the negative chronotropic effect of ET-1 [261,262]. At the same time, ETB receptors, mainly located on endothelial cells, mediate vasodilation by inducing NO production through eNOS [263]. NO synthesis also mediates endothelin-stimulated migration of endothelial cells during the formation of new vessels [264,265]. In this case, NO induces the transition from a stationary endothelial cell phenotype to movement, and the guiding signals are provided by VEGF, which provides the vector component of endothelial cell movement [266].

In addition to its involvement in the regulation of vascular hemodynamics, endothelin-1 has been actively involved in cross-talk with inflammation. Endothelin-1 increased the expression of genes involved in eicosanoid biosynthesis (*Pla2g4a*, *Pla2g4b*, *Ptgs2*, *Ptgis*, *Alox12*, and *Alox15*) in the rat experiment of [267]. It also induced the release of arachidonic acid through the activation of cytosolic phospholipase A2 in rat vascular smooth muscle [268]. Endothelin-1 released eicosanoids from the isolated perfused kidney (more PGI2 than PGE2 but not TXA2) and spleen (mainly PGE2 and, to a lesser extent, PGI2 and TXA2) of the rabbit [269]. Endothelin-1 also released EDRF from the endothelium of a perfused rabbit aorta [270]. ET-1 caused dose-dependent release of PGE2, 6-oxo-PGF1 alpha, and TXB2 into splenic venous effluent, with endogenous prostanoids modulating endothelin-1 vascular responses [271].

ET-1 is a strong chemoattractant for blood monocytes [272]. ET-1 has been shown to stimulate monocyte release of IL-8 and MCP-1, which are chemoattractants for neutrophils and monocytes [273]. In addition, endothelin-1 stimulates monocyte production of TNF-α, IL-8, granulocyte–macrophage colony-stimulating factor, IL-1β, and IL-6 [274,275].

Thus, endothelin-1 may be involved in endothelial dysfunction by regulating inflammation and vascular remodeling. Levels of ET-1 are elevated in patients with atherosclerosis. In this regard, endothelin-1 is considered a marker of disease and a predictor of the severity and prognosis of coronary heart disease.

### 3.2. Other Innate Immune Functions of the Endothelium

A growing body of evidence allows us to identify endothelial cells as conditional innate immune cells [276]. As some of the first cells to detect endogenous agents and foreign pathogens in the bloodstream, endothelial cells function as sensors of danger signals. To this end, endothelial cells express innate immune system receptors such as TLR and NLR and also express chemokine receptors [277,278,279,280]. In addition to the expression of the receptors themselves, endothelial cells express important downstream adaptor molecules for pattern recognition receptors (PRR) signaling, including MD2 and MyD88 [276,281].

Endothelial Toll-like receptor 4 (TLR4) demonstrates an important cross-talk between disturbed blood flow and inflammation [282]. It has been shown that under chronic laminar blood flow, human coronary artery endothelial cells (HCAECs) are hyporesponsive to stimulation by specific TLR2 ligands. This is because of laminar flow-induced phosphorylation of serine SP1 by CK2 protein kinase in HCAECs. This results in blocking of the binding of SP1 to the TLR2 promoter, which is essential for TLR2 expression [283]. This mechanism provides inhibition of TLR2 upregulation in human endothelial cells by LPS and TNF [283].

NLRP3 inflammasome activation is involved in endothelial dysfunction, exacerbating subsequent inflammatory cascades and cell damage [284]. Activation of the NLRP3 inflammasome initiates the production of mature forms of IL-1β and IL-18 from cells, contributing to further inflammation and oxidative stress in the endothelium [284].

In the quiescent state, endothelial cells do not interact with leukocytes but increase the expression of adhesion molecules and chemokines during inflammation, leading to transmigration of leukocytes through the endothelium to the site of inflammation [285].

In addition, like cells of the innate immune system, when a pathogen is detected, endothelial cells produce cytokines and chemokines, which attract phagocytes to the site of infection. Endothelial cells have been shown to secrete the proinflammatory IL-8 via NOD1/NF-kB in response to microbial stimulation [286]. Endothelial cells infected with *Staphylococcus aureus* displayed IL-8 expression, which may promote neutrophil transmigration through the endothelium [287].

In addition to participation in the presented mechanisms of the innate immune system, endothelial cells function as antigen-presenting cells. In response to inflammatory stimuli, endothelial cells can transdifferentiate into antigen-presenting cells by expressing major histocompatibility complex class II (MHC-II) molecules and T-cell costimulation/coinhibition molecules [288].

In addition, endothelial cells were found to have potential phagocytic capacity, being nonprofessional phagocytes [284]. Endothelial cells can phagocytize bacteria through non-opsonic and opsonic mechanisms [289].

Endothelial cells have been shown to use a formin-dependent process, similar to phagocytosis, to internalize *Listeria monocytogenes* bacteria [290]. Endothelial cells have also been found to kill non-virulent strains of phagocytosed *Staphylococcus aureus* [291]. In addition, in patients with essential thrombocythemia, endothelial cells perform phagocytic clearance of platelets, which represents a new mechanism for removing activated platelets from the bloodstream [292]. Endothelial cells can also influence inflammation through phagocytosis of senescent neutrophils [293]. Interleukin-1 has been shown to enhance binding and endocytosis of apoptotic bodies by liver endothelial cells [294].

Another mechanism of involvement in the innate immune system is the expression of antimicrobial peptides, as shown in experiments with bovine endothelial cells [295]. Human umbilical vein endothelial cells also actively contribute to preventing the spread of pathogens through the blood–tissue barrier by producing antimicrobial peptides that exhibit bactericidal and immunomodulatory functions [296]. The human antimicrobial peptide β-defensin-3 has been shown to accelerate wound healing by promoting angiogenesis, cell migration, and proliferation through the FGFR/JAK2/STAT3 signaling pathway [297].

Thus, endothelial cells share some characteristics with macrophages and neutrophils in that they have phagocytic and motile properties [298]. The ability of endothelial cells to align and migrate under the influence of hemodynamic factors seems interesting [299,300]. Lymphatic microvascular endothelial cells have been shown to migrate upstream against the flow direction [301]. However, upstream cell migration was observed in the presence of intercellular contacts, whereas cells with few contacts migrated downstream [301]. In another study, it was also shown that microvascular endothelial cells at high cell density migrate against the direction of the fluid flow and concentrate in the region of maximum shear stress. In contrast, microvascular endothelial cells at low density (isolated), which lack intercellular contacts, migrate in the direction of the flow [302]. Thus, noncontact human umbilical vein endothelial cells (HUVECs) migrate downstream in the vascular stream [302]. The shear stress distribution with large gradients created by oncoming flow stimulates HUVECs to migrate against the flow direction and into the region of maximum shear stress [302]. Interestingly, sPLA2 enhanced bovine aorta endothelial cells’ (BAECs) migration, which is related to its involvement in arachidonic acid release [303]. Arachidonic acid at low concentrations promotes cell migration through a mechanism involving products of metabolism of this compound [304]. At the same time, higher concentrations of arachidonic acid led to more random and less directed movements of individual cells, which may be due to the incorporation of arachidonic acid into plasma membranes, with a corresponding change in their physical properties, which may have an inhibitory effect on cell motility [305].

Thus, endothelial cells demonstrate a variety of functions in the vascular wall. The function of hemodynamic regulation in this case is closely related to the participation of endothelial cells in the innate immune system, and these cross-links have deep evolutionary roots.

## 4. Conclusions

Endothelial cells are a heterogeneous population of cells that perform many physiologically important functions. As part of the barrier between the blood and tissues of the body, endothelial cells participate in the functioning of this barrier by regulating its permeability to molecules and cells. This function also ensures the maintenance of arterial pressure and is closely related to the participation of endothelial cells in the regulation of vascular hemodynamics. Many endothelium-dependent mechanisms are known to be involved in the regulation of vascular tone. There is also ample convincing evidence that mechanisms of hemodynamic regulation are closely related to mechanisms involved in inflammation in the vascular wall. Many of these mechanisms have evolutionarily conditioned cross-linkages, the study of which will improve the understanding of vascular function and its disorders in atherosclerosis.

Importantly, endothelial cells demonstrate involvement in many mechanisms of the innate immune system, abnormalities of which are associated with the development of atherosclerosis. Endothelial cells modulate inflammation by regulating immune cell transport, activation status, and function.

Thus, endothelial cells are at the crossroads between the hemodynamic characteristics of blood flow and the innate immune system. These connections have deep evolutionary roots and may be disrupted during atherogenesis.

## Data Availability

Not applicable.
